# Central Commands to the Elbow and Shoulder Muscles During Circular Planar Movements of Hand With Simultaneous Generation of Tangential Forces

**DOI:** 10.3389/fphys.2022.864404

**Published:** 2022-05-19

**Authors:** Alexander I. Kostyukov, Andriy V. Gorkovenko, Yurii A. Kulyk, Oleksii V. Lehedza, Dmytro I. Shushuiev, Mariusz Zasada, Serhii S. Strafun

**Affiliations:** ^1^ Department of Movement Physiology, Bogomoletz Institute of Physiology, National Academy of Sciences, Kyiv, Ukraine; ^2^ Department of Physical Education, Gdansk University of Physical Education and Sport, Gdansk, Poland; ^3^ Institute of Traumatology and Orthopedics, National Academy of Medical Sciences of Ukraine, Kyiv, Ukraine; ^4^ Faculty of Physical Education, Health and Tourism, Institute of Physical Culture, Kazimierz Wielki University, Bydgoszcz, Poland

**Keywords:** forelimb, motor control, two-joint movements, muscle synergy, motor commands, electromyogram

## Abstract

This study examines some of the non-linear effects of signal transduction in the human motor system, with particular emphasis on muscle hysteresis. The movement tests were analyzed in a group of eight subjects, which were asked to develop tangential force using visual biofeedback while performing slow, externally imposed, circular movements of right hand holding a moving handle operated by a computerized mechatronic system. The positional changes in the averaged EMGs of the elbow and shoulder muscles were compared for all combinations of direction of movement and generated force. Additionally, for one of the subjects, there was carried out MRI identification and 3D printing of the bones of the forelimb, shoulder, scapula and collarbone, which made it possible to reconstruct for him the length and force traces of all the muscles under study. The averaged EMG traces in muscles of both joints show their close correspondence to the related force traces, however, the co-activation patterns of activity in agonists and antagonists were also often encountered. The EMG waves related to the respective force waves were strongly dependent on the predominant direction of the muscle length changes within the correspondent force wave locations: the EMG intensities were higher for the shortening muscle movements (*concentric* contractions) and lower during muscle lengthening (*eccentric* contractions). The data obtained allows to suggest that for two-joint movements of the forelimbs, it is sufficient to consider the *force* and *activation synergies* (patterns of simultaneous activity in different muscles), ignoring at the first stage the effects associated with *kinematic synergy*. On the other hand, the data obtained indicate that the movement kinematics has a strong modulating effect on the *activation synergy*, dividing it into *concentric* and *eccentric* subtypes, in accordance with the known non-linear features of the muscle dynamics. It has been shown that the *concentric* and *eccentric* differences in the responses of the shoulder muscles are more clearly distinguishable than those in the elbow muscles. The shoulder muscles also have a more pronounced symmetry of the averaged EMG responses with respect to the ascending and descending phases of force waves, while demonstrating a lower degree of antagonist cocontraction. The data obtained suggest that the central commands in two-joint movements are determined mainly by the interdependence of *force* and *activation synergies* including both intra- and inter-joint components, while *kinematic synergy* can be interpreted as a potent modulator of *activation synergy*.

## Introduction

The concept of “synergy,” as intended in the analysis of purposeful human movements, including the shared behavior of a large number of muscles, allows for various formulations. The most common formulation is the traditional, rather philosophical, designation of synergy for describing complex purposeful motor acts (for review, see Latash, 2021). This concept is closely connected with the problem of redundancy of the motor control system introduced by Bernstein in his hierarchical theory of voluntary movement (Bernstein, 1967). In Bernstein’s theoretical studies, the idea of synergy is considered the ability of the central nervous system (CNC) to eliminate the kinematic redundancy of motor acts by reducing the excessive degrees of freedom (DOF) in the programs of movement. The degrees of freedom are not considered elements of (bio)mechanics here, so the above mechanism can be interpreted as a hypothetical elimination of the redundancy of commands sent by CNC to the pool of synergistically interacting muscles. For example, such an approach to movement analysis has been shown for complex control signals to a “frozen” wrist joint when a pure elbow movement is generated (Latash et al., 1999). A number of computational algorithms have been proposed to evaluate the effectiveness of human movement control using a limited number of motion primitives; some of these algorithms include principal component analysis, independent component analysis, factor analysis, and nonnegative matrix factorization (Saltiel et al., 2001; Hart and Giszter, 2004; Tresch et al., 2006). Various problems in the analysis of movement control using a limited number of muscle synergies have been described in a few review articles (Tresch and Jarc, 2009; Safavynia et al., 2011; Bizzi and Cheung, 2013; Giszter, 2015).

The stability of synergies in motor actions convincingly confirms the existence of this concept of motor control in humans. Accordingly, various studies have confirmed the possibility of such control mechanisms in movements of the upper extremities in humans (d'Avella et al., 2006; d'Avella and Lacquaniti, 2013). It has been demonstrated that muscle synergy can probably reconstruct muscle activity during virtual trajectories of arm movement or force generation, and the synergy models can sometimes satisfactorily reconstruct variations in EMG data (Berger et al., 2013).

**FIGURE 1:**
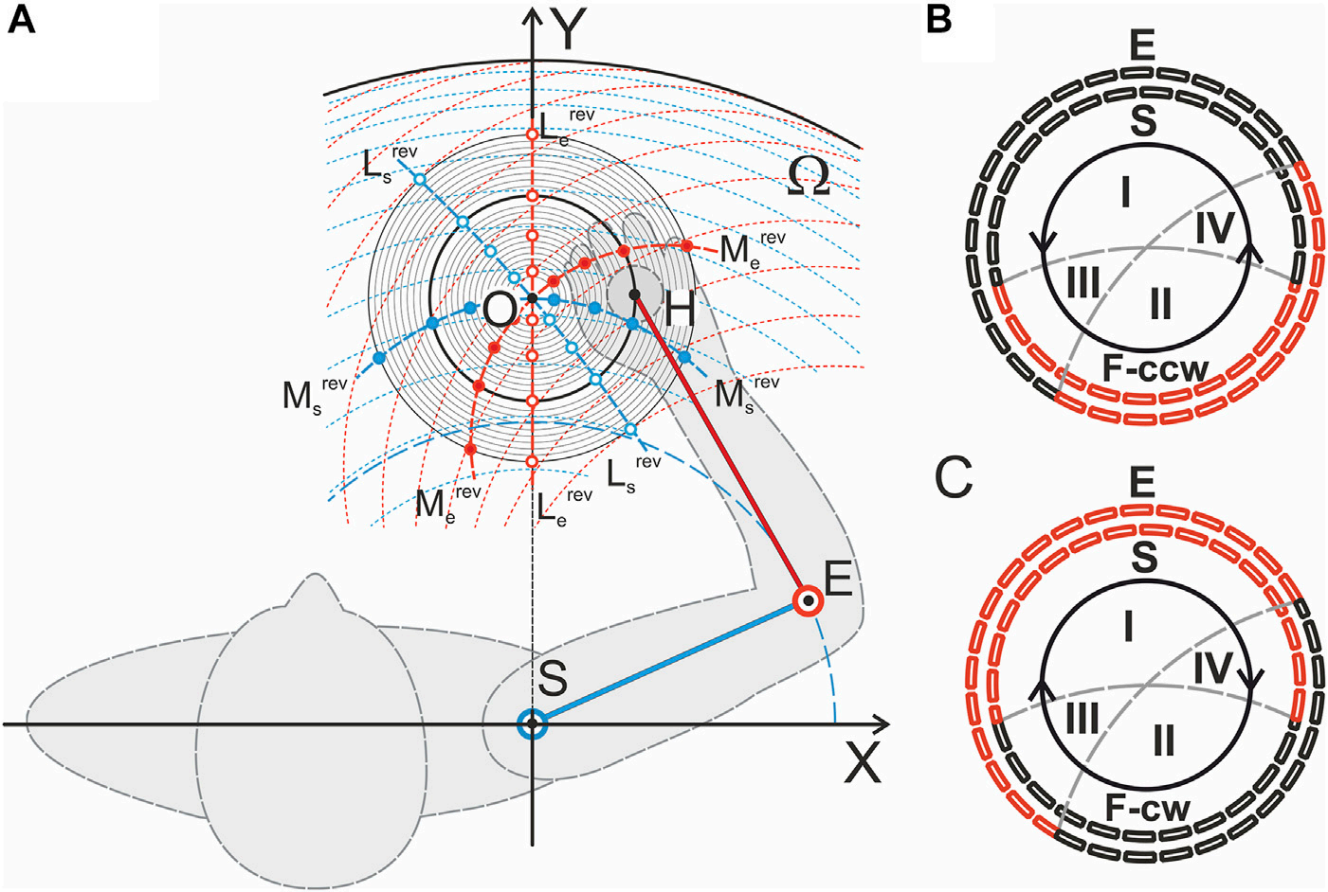
Experimental setup. **(A)** Geometry of the test movements; the respective graphs are built in a scale for the subject YK, for whom the tomographic identification of the joint bones had been preliminarily performed (Gorkovenko et al., 2020). The center of the circle of movement (a thickened line in the family of concentric circles, R = 10 cm) was located on a line perpendicular to the frontal plane passing through the axis of the subject’s right shoulder joint; the distance from the circle center to the joint center was 40 cm, and the lengths of the SE and EH segments were 28 and 32 cm, respectively (the distances can be used as a scale for this Panel). The red dashed lines represent traces of movement in the elbow joint for a fixed set of shoulder positions; the blue dashed lines show movements in the shoulder joint during stepwise fixation of the elbow joint angle. Additionally, the *reverse lines* for the torques acting around the shoulder and elbow joints (M_s,e_^rev^) and for the directions of the muscle length changes (L_s,e_^rev^) (Kostyukov, 2016) are shown in **(B,C)**. The segments of the circular movement where the flexor (black) and extensor (red) muscles belonging to the shoulder (E, outer dashed circles) and elbow (S, inner dotted circles) joints actively contract to develop tangential forces in counterclockwise F (ccw) or clockwise F (cw) directions are shown. Synergy sectors I and II define the zones in which combinations of the elbow and shoulder muscles of the same functionality (flexor-flexor, extensor-extensor) are activated to create tangential forces; in sectors III and IV, the flexor-extensor or extensor-flexor pairs should be active. A change in the force direction, F (ccw) on F (cw), and vice versa leads to a change in the positions of the activation sectors between the flexors and extensors of both joints.

Experimental and analytical problems for the study of complex voluntary movement acts, including many joints and muscle groups, can be somewhat smoothed for simpler experimental movements, such as two-joint planar arm movements. Recent studies by our group have been devoted to the search for synergistic patterns of activation of muscles belonging to the elbow and shoulder joints (Abramovich et al., 2015; Kostyukov, 2016, 2019; Tomiak et al., 2016; Kostyukov et al., 2019). This approach allows the production of rather accurate, repeatable movements using visual control of the end-point trajectories (Abramovich et al., 2015; Tomiak et al., 2016). In the present study, we propose a new approach to study two-joint forearm movements using a computerized mechatronic mechanostimulator (MMS). When a subject catches by his hand the moving handle of MMS, this device allows him to reproduce prescribed planar two-dimensional hand movements; here, we consider slow circular movements of changing direction (Figure 1). In addition to the program of passive hand movement imposed by MMS, the subject, using the visual biofeedback mode, creates a varying force pressure on the manipulator handle with his hand; the direction of the force vector command changes in accordance with the current position of the hand during movement while maintaining its tangential direction. Thus, MMS provides separate programming of the “passive” and “active” components of the movement.

The geometrical modeling of the two-joint planar arm movements considers the distribution of various types of force-dependent synergies in the possible central activation patterns of the muscles of the proximal and distal joints (Kostyukov, 2016). The study considers the linear and circular movements of the end-point (hand) and cases of two opposite forces applied tangentially with respect to the end-point trajectory. Additionally, theoretical approaches were elaborated for quantitative modeling of the efferent commands that should be created by the central nervous system to provide a given trajectory of the muscle length change under the action of a given temporal profile of the external force loading the muscle (Kostyukov, 2019). Compared to the simplified hinge joints used to geometrically model arm movements, synovial joints not only exhibit isolated rotations but are also capable of exhibiting some displacement movements. It is known that the amplitudes of translational motion in the shoulder joint can extend up to several cm during large-scale movement; therefore, the segments of the human hand cannot be properly represented as a rigid body fixed at a certain point (Israely et al., 2018). In any case, to increase the accuracy of the quantitative analysis of real movements, we need precise knowledge of the main anatomical parameters of the muscles participating in the movements. First, this concerns assessments of the muscle lengths and the shoulders of force moments and their change during movement. To make a real step in this direction, for one of the subjects, there was carried out MRI identification and 3D printing of the arm bones, which made it possible to reconstruct the length and force traces of all the muscles under study. (Gorkovenko et al., 2020).

The main objectives of this study are to analyze in detail the central commands that govern the execution of circular movements of the hand in the horizontal plane with parallel creation of the end-point force directed tangentially to the movement trajectory. There are four possible combinations in the directions of movement and force. Particular attention is given to the analysis of various types of muscle hysteresis, showing the dependence of the state of the muscle on the previous history of activation and movement. Hysteresis can be represented in 3D form as the dependence of averaged EMG records on changes in muscle length and force (Kostyukov et al., 2019). Various types of synergies between the central commands accompanying the execution of the circular movements are discussed with a special attention to the geometry of test movements (Figures 1B,C). Preliminary results were published earlier (Tomiak et al., 2016), and the present study was performed using a new experimental setup (Zasada et al., 2020) and anthropometric identification of the arm bones in one of the study participants (Gorkovenko et al., 2020).

Hypothesis. The force and activation synergies can be considered the main elements describing the circular two-joint movements (elbow, shoulder) of the human forelimb. It is assumed that activation synergy includes the intra- and inter-joint components, the first of which determines the similarity of activation patterns in groups of agonist and antagonist muscles belonging to each of the joints; the second one reflects the features of the simultaneous activation of muscles of different joints. The role of the movement kinematics is to modulate the activation synergy, dividing its effects into concentric and eccentric subtypes.

## Methods

Eight adult right-handed men (aged 21–29 years old, mean 26.2 ± 3.8) participated in the experiments. The experimental procedures used were in accordance with the ethical standards of the research committee of Bogomoletz Institute of Physiology, National Academy of Sciences, Kyiv, Ukraine, and with the 1964 Helsinki declaration and subsequent amendments or comparable ethical standards. Informed written consent was obtained from all participants. The experimental procedure did not exceed 1.2 h. The mechanical disposition of the subjects within the setup is schematically presented in Figure 1A; detailed patterns of the reverse lines for the torques acting around the joints (M_s,e_^rev^) and for the directions of the muscle length changes (L_s,e_^rev^) (Kostyukov, 2016) are shown in Figures 1B,C.

### Robotic-Mechatronic Device for Creating Imposed Movements of the Subject’s Arm

The mechanostimulation technique is described in detail in our earlier paper (Zasada et al., 2020). The respective device is based on a modern robotic system that controls 2D planar transitions of the MMS handle; similar devices serve as 3D printer components. A flat basement of the MMS was fixed horizontally in such a position that the handle of the manipulator, installed vertically at the moving platform, was located approximately at the level of the center of the subject’s shoulder joint (Figure 1A). The circular trajectories of the imposed movement were used in the present study. The subject clasped the handle with his right hand, and the arm was additionally supported at the elbow by a wide cloth loop, which was attached to the ceiling with a long rope to reduce unnecessary muscle activity so that the horizontal position of the arm could be maintained. While holding the MMS handle, following the movement of the manipulator, the subject passively performed the assigned imposed movement program. The movements of the MMS handle were determined by the work of two linear drives, the movable carriages of which were driven by the stepper motors. The linear drives were located perpendicular to each other; the horizontal drive (defining the abscissa of the MMS handle position) was fixed on the MMS basement (a table), while the vertical drive (ordinate) was rigidly attached from above to the movable carriage of the former drive. Switching on/off and the rotation velocity of the stepper motors (consequently, the position of the manipulator handle) were controlled by a computer using MACH 3 software (ArtSoft, United States). Registration of the position of the hand on the handle of the manipulator in Cartesian coordinates, the beginning of which coincided with the axis of rotation of the patient’s shoulder joint, was carried out using precision potentiometric sensors.

### Device for Recording the Force Vector Created by the Subject’s Hand

The device presented the rod, installed vertically at the moving platform, as a part of the manipulator handle of the MS. The rod was assembled from two identical standard load cells rigidly fixed to each other along the longitudinal axis in such a way as to ensure the perpendicular arrangement of the sides of the preferred deformation. A detailed description of the device can be found in the study cited above (Zasada et al., 2020); it allows accurate measurement of the amplitudes and directions of the force vectors applied to the manipulator handle in any direction on the horizontal plane.

### Visual Biofeedback System for the Arbitrary Contraction Program

The combination of a force vector recording device, visual biofeedback and mechatronic MMS allows us to combine the imposed movement program (IMP), i.e., a hand movement along a given trajectory, with an arbitrary contraction program (ACP), which informs the subject about the reproduction of the required force. In this study, the ACP is prepared in such a way that the command signal in the form of a luminous point on the monitor screen moves along a circular path that corresponds to the actual path of the subject’s hand. If the subject satisfactorily reproduces the rotation of the force vector by combining its end point with a moving control point on the monitor, then the vector changes continuously as it moves. If the turning angle changes in a counterclockwise direction [T(ccw)], then to create the tangential force in the same [F(ccw)] or opposite direction [F (cw)], the subject must change the vector of his force along a similar circular path, which either goes ahead or lags by 90° behind the movement trajectory. Similar relationships are valid for the clockwise direction of the turning angle [T(cw)].

Thus, a program in which the voluntary contraction of the “target” arm muscles in accordance with the ACP was closely combined with the IMP. The MMS possessed sufficient mechanical stiffness (high mechanical impedance) so that no additional displacements of the MMS handle occurred within a certain range of the applied forces.

### EMG Recording and Data Processing

The surface EMGs were registered from the following eight muscles: *mm. pectoralis pars clavicularis* (Pect), *deltoideus pars scapularis* (DeltSc), *deltoideus pars clavicularis* (DeltCl), *biceps brachii caput longum* (BicLg), *biceps brachii caput breve* (BicBr), *brachioradialis* (BrRad), *triceps brachii caput laterale* (TrLat), and *triceps brachii caput longum* (TrLg); the abbreviations used throughout the remainder of the paper are given in brackets. The EMGs were recorded by pairs of electrodes (Biopac System EL 503, United States) with a center-to-center distance near 25 mm. The electrodes were fixed on the subject’s right arm over the belly of the muscles. The recorded activities were amplified via a 16-channel amplifier (CWE, Inc., PA 19003 United States) and filtered in the range of 10–5,000 Hz. The EMGs together with the position signals from the mechatronic device were collected via a CED Power 1401 data acquisition system using the Spike 2 (Cambridge Electronic Design, United Kingdom) program. The amplified signals were digitized at 10 Hz, and Origin 8.5 (OriginLab Corporation, United States) was used for the off-line data analysis. The EMG records were full-wave rectified and filtered (Batterworth filter of fourth order, bandwidth 0–10 Hz) in an off-line regimen; this procedure introduced a phase lag with respect to the real changes in the EMG intensity near 130–150 ms; the angle errors for the EMG-turning angle presentations used did not exceed ±2.2°. All tests were repeated 6–8 times to obtain the average corresponding records. At the end of each experiment, we registered the maximal voluntary contraction (MVC) of each muscle undergoing study. For this purpose, the averaged EMG levels during steady state maximal isometric contractions of the muscles when the shoulder and elbow angles were near 70 and 90°, respectively, were defined. Similarly, the minimal levels of EMG activity in fully relaxed muscles were evaluated. The average EMG activity registered in the main part of the experiments is shown in the percentage scales, which ranged from the above-defined minimal levels of activity (0%) to the MVCs (100%).

### Evaluation of Changes in Muscle Length and Acting Force

The muscle length evaluation was based on the measurement of a graphic model built on images of upper extremity bones and soft tissue. Here, we provide only a concise description; however, more details can be found in our earlier publication (Gorkovenko et al., 2020). Images of the bones of the right arm (clavicula, scapula, humerus, ulna and radius) were obtained by X-ray tube. Images of soft tissue were obtained by the MRI method. The models of all scanned bones were printed using a 3D printer, and two independent experts, a surgeon and a traumatologist, marked the areas of exit and attachment of the main brachial grid and arm muscles on these 3D models of bones in accordance with the computer scan. At the next stage, the bones of the limbs were graphically modeled in various positions with different joint angles. Then, the surgeon and traumatologist placed the virtual muscles in the appropriate positions according to their previously determined origin and insertion areas, computed tomography scans, and anatomical atlases. Such plots were made for values in the shoulder joint angle from –20–120° with a 10° step and in the elbow joint from 0 to 130^°^. The muscle length was measured along the midline of the muscle projection for all measured arm configurations. Additionally, the force arms were evaluated for the corresponding muscles. Based on the measurements, the respective regression formulae were determined for changes in the lengths of muscles and their arm forces dependent on the angles in the joints. The general expressions are presented as follows:

$$L=Z_{0}+\sum_{n=1}^{3}A_{n}.\varphi_{s}^{n}+\sum_{n=1}^{3}B_{n}.\varphi_{e}^{n},$$ (1)

where Z, A, and B are the regression coefficients, and $\phi_{s}$ and $\phi_{e}$ are the shoulder and elbow joint angles in degrees.

To evaluate the muscle forces, the joint torques were first determined (Gorkovenko, 2018). The joint torques were determined by a system of standard classic mechanics equations corresponding to the real positions of the arm segments along the movement trajectory. Then, the simplified model, in which the joint torques were created by only a single agonist muscle excluding the action of other agonists and antagonists. In this case, the corresponding force *F* is defined as follows:

F=M/l, (2)

where *M* and *l* are the joint torque and force arm, respectively.

In our earlier EMG studies with different types of planar arm movements (Tomyak et al., 2016; Kostyukov et al., 2019), we failed to trace the exact trajectories of changes in the lengths of the studied muscles because of a lack of respective biomechanical information, it was also impossible to accurately determine the forces produced by the various muscles. On the other hand, such information becomes especially important when trying to model the dependency of the central commands to muscles on changes in their forces and lengths during movements (Kostyukov, 2019). Unfortunately, the high cost of the tomographic identification method did not allow this approach to be applied to all subjects participating in this study.

### Statistical Analysis

Statistical analysis was applied to the EMG activities of the muscles under study. All statistical computations were performed using the programs Origin 8.5 (OriginLab Corporation, United States) and SPSS 17.0 (IBM Business Analytics software, United States). A two-sample *t* test was used to determine the statistical significance of the difference between the respective parameters.

## Results

### A General Description of Experiments

**FIGURE 2:**
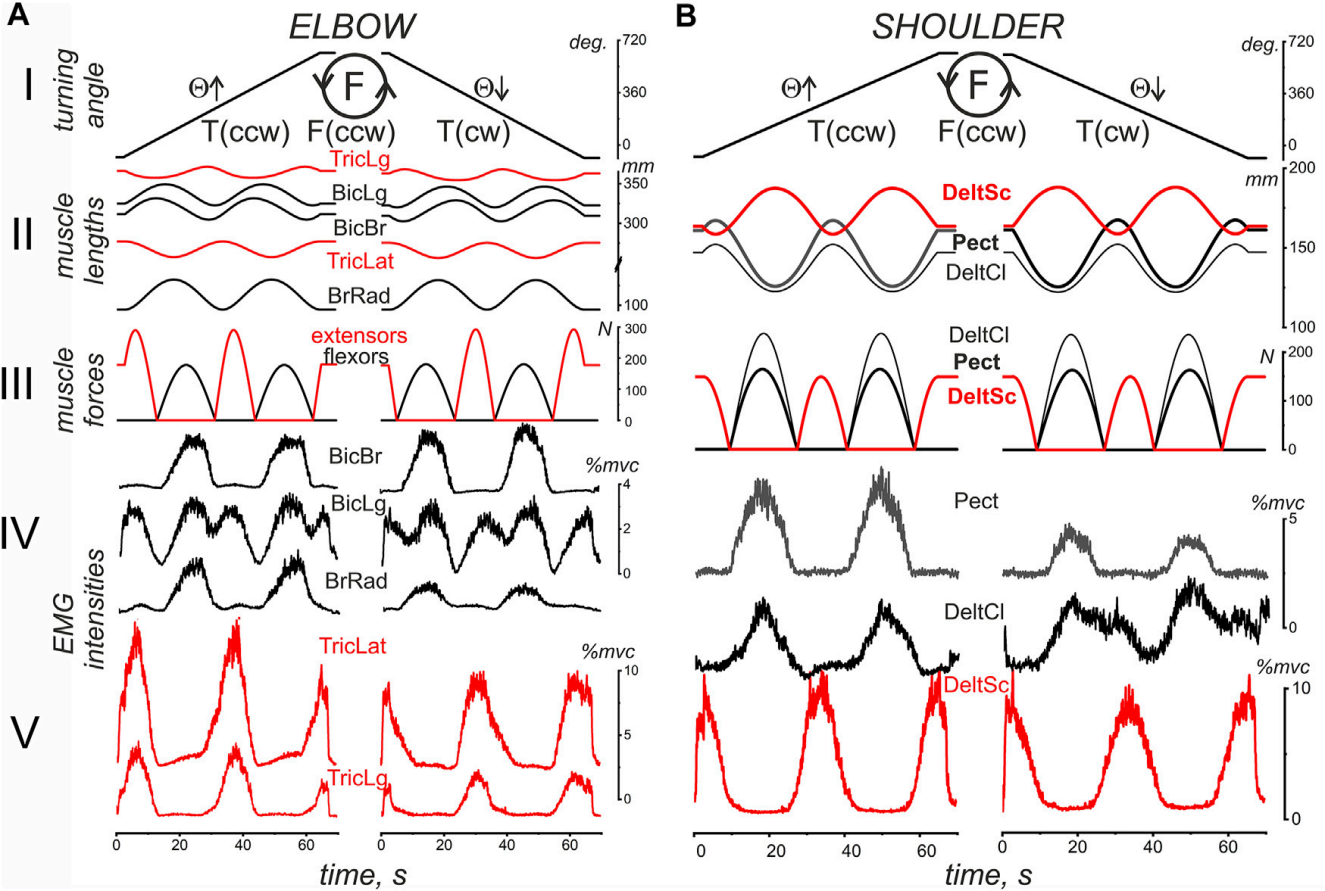
An example of typical recordings of EMG activity in the flexor (black) and extensor (red) muscles (subject YK with identified bone geometry). The muscles belong to the elbow **(A)** and shoulder **(B)** joints during standard test movements, including the creation of counterclockwise hand force (F inside the drawn circle); the same movement program (not shown in this panel) is then repeated for a clockwise direction of force. The movement trajectory of the handle under the subject’s right hand (H), which has been defined by the program of the related turnings of the step motors, is presented as the turning angle (Θ) of the handle’s position at the circular trace; the EMG reactions during the rising (Θ↑) and falling (Θ↓) branches of movement are considered separately. The pressure on the handle was created by a subject in accordance with an arbitrary contraction program (ACP) that was prepared in advance together with the imposed movement program (IMP) for the handle transitions of the mechatronic mechanostimulator (MMS). The muscle lengths and forces were evaluated in an off-line regimen using a corresponding model approach based on the 3D prints of the bones of this subject (Gorkovenko et al., 2020), therefore the presented single records of force and length correspond to all identical movements used for further averaging procedure of the EMG records in six repetitions of the tests (see Methods). Note the different calibration scales for EMGs recorded from different muscles.

A general scheme of the experimental procedure is shown in Figure 2. The IMP was started at the turning angle Θ = −90°; then, a slow uniform movement (Θ↑) continued for approximately 60 s while turning the manipulator handle in counterclockwise direction, T(ccw); to the end of this time interval, two whole rotation cycles were completed. Movement was stopped at Θ = 630° for 20 s; then, the turning continued in the reverse direction [T (cw), Θ↓] until arriving at the start position (Θ = −90°). During IMP, a subject produced the forced pressure on the manipulator handle in accordance with ACP. Figure 2 shows typical changes in muscle length and strength defined offline (see METHODS) in parallel with the online turning angle traces. Both the muscle lengths and force trajectories are symmetric with respect to the direction of change of the turning angle. However, the symmetry of the single force waves in the flexors and extensors of both joints coincides with the asymmetric arrangement of the ascending and descending phases of the changes in their lengths. Such an arrangement of force waves at the lengthening and shortening phases of the change in length causes a noticeable difference in the corresponding EMGs in the T (ccw) and T (cw) sections of movement. Force waves in all muscles of both joints mainly coincide with their shortening (lengthening) on the left (right) part of the movement traces. The actively contracted muscles need a more powerful efferent inflow during their shortening; thus, the corresponding EMG reactions in most cases are more pronounced at the T (ccw) movements (left halves of Panels A, B in Figure 2); this can be observed in the reactions of *BrRad, TricLat, TricLg, Pect, and DeltSc*. On the other hand, not all the muscle activity involved in movement was recorded; therefore, the joint interactions of many muscles, both agonists and antagonists, can cause effects that do not fit into a simple scheme. At least in part, some abnormalities could be observed in the *BicBr* and *DeltCl* responses; moreover, a less obvious relationship between the respective force waves and EMG responses was also present in this experiment in the *BicLg* responses, showing obvious coactivation with antagonistic muscles.

We would like to stress that the length traces of muscles belonging to the elbow joint (Figure 2A) do not fully correspond to the simplified geometric model of the two-joint movement in Figure 1 that assumes a rigid coincidence of the reverse points for all muscles of each joint. Note that the muscle length trajectories in Figure 2 were identified by the realistic arm models using the 3D MRI-reconstructed bone prints of the subject. Significant deviations were observed between changes in the length of the bi and monoarticular muscles, at least when comparing the muscle pairs *BicLg* and *BicBr*, as well as *TricLg* and *TricLat* (Figures 2AII). The first muscles in these pairs are monoarticular, and their lengths depend on the angles of both joints, while the lengths of the second muscles depend only on the elbow joint angle. On the other hand, the muscles of the shoulder joint are monoarticular; therefore, their reverse points coincide (Figure 2B).

### Comparison of the Average EMG Activities in the Flexor and Extensor Muscles

**FIGURE 3:**
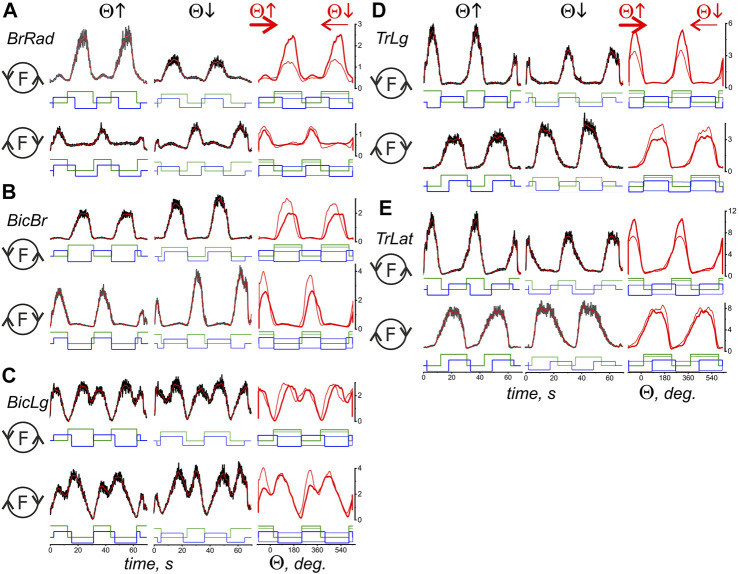
Comparison of the average EMG activities in the flexor and extensor muscles of the elbow joint during standard test movements produced during the creation of the hand force in opposite directions. The data were obtained of subject YK with identified bone geometry, repetition of six identical tests for averaging procedure. The EMG reactions on the rising (Θ↑) and falling (Θ↓) branches of the turning angle change are compared, and the circle with the letter F inside designates the force direction in the respective movement tests. The first two columns present the time scale, thus including the averaged EMG records (black) with subsequent filtering by 300-point Savitsky-Golay smoothing (red lines); the filtered records are additionally transformed to depend on the turning angle and are superimposed in the third column; the thick and thin red lines correspond to the rising and falling branches of the turning angle, respectively. The stepwise records below the EMGs describe the timing of both the respective force waves (green) and the directions of the muscle length changes (blue). The “up” and “down” on the blue records correspond to the lengthening and shortening movement phases, respectively. The thick EMG records in the third column evolve from left to right, whereas the thin records evolve in the opposite direction. The EMG intensities are calibrated based on % of the corresponding MVC values. A continuation of the presentation of this experiment for the shoulder muscles is shown in Figure 4 **.**

**FIGURE 4:**
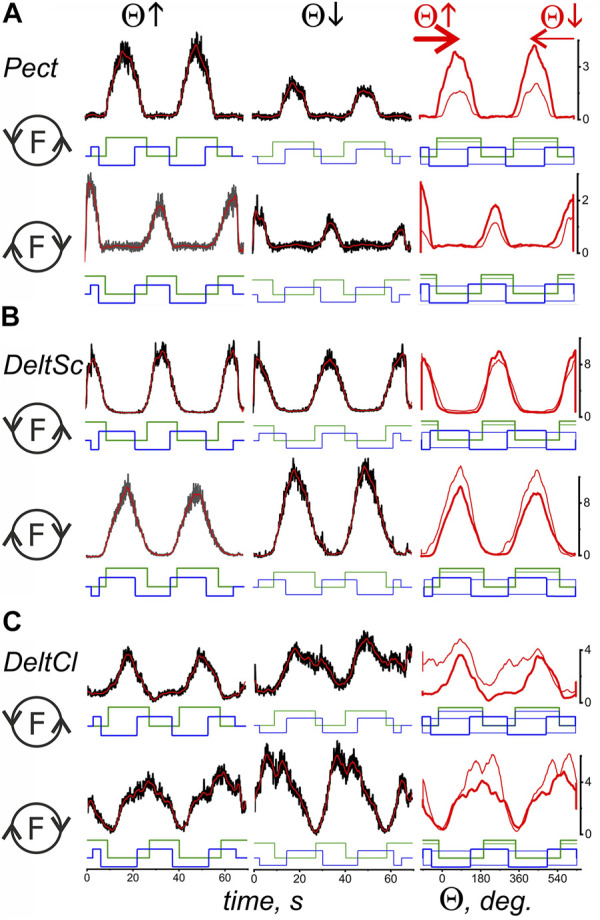
Comparison of the averaged EMG activities in the flexor and extensor muscles of the shoulder joint during test movements. The data were obtained in the same experiment as in Figure 3; combining both drawings proved impractical due to their scope. The structure of the plots and all the designations (except for the names of muscles) are the same as in Figure 3.

Figures 3, 4 compare EMG responses when the generated forces are applied in opposite directions from each other. The preprocessed EMGs were additionally filtered using 300-point Savitsky-Golay smoothing (red lines overlaid on the row EMG records). To simplify the information about the mechanical events in these tests, only switching times are shown instead of the force and length records (green and blue lines, respectively). Figure 1, which describes the geometric model of the circular arm movements, shows that a change in the direction of the tangential force generated by the hand leads to a change in the order of activation of the flexor and extensor muscles of both joints. When comparing flexor and extensor contractions in the same joints, such as *BrRad* and *TrLg* (Figures 3A,D) and *Pect* and *DeltSc* (Figures 4A,B), the force and respective EMG waves in the flexors are longer for F (ccw) and in the extensors for the F (cw) force directions. The temporal positions of the EMG reactions correlate with the positions of the force waves; after changing the direction of the tangential force, the ratio between the duration of the waves in the flexors and extensors is reversed.

A reversal of the movement direction produced by the transition from T(ccw) (Θ↑) to T(cw) (Θ↓) evokes similarly directed changes in the EMG amplitudes in the flexor and extensor muscles of both joints, i.e., *BrRad* and *TrLg* (Figures 3A,D) and *Pect* and *DeltSc* (Figures 4A,B); the differences in EMG reactions can be observed more clearly when they are superimposed on each other depending on the turning angle (thick and thin red lines in the third columns of Figures 3, 4). However, exceptions in these patterns can sometimes occur; for example, in the present experiment, this is observed for *BicBr* when F (ccw) forces are applied (Figure 3B, top part); there is also no clear difference between the EMGs in *BicLg* (Figure 3C, top part). The differences in EMGs are more evident in the elbow extensors (Figures 3D,E), as well as in the muscles of the shoulder (Figure 4); however, in this case, some deviations can also appear. For example, this may apply to the EMGs of *Pect* and *DeltCl* (Figures 4A,C).

### Hysteresis Effects in Elbow and Shoulder Muscle Activity

**FIGURE 5:**
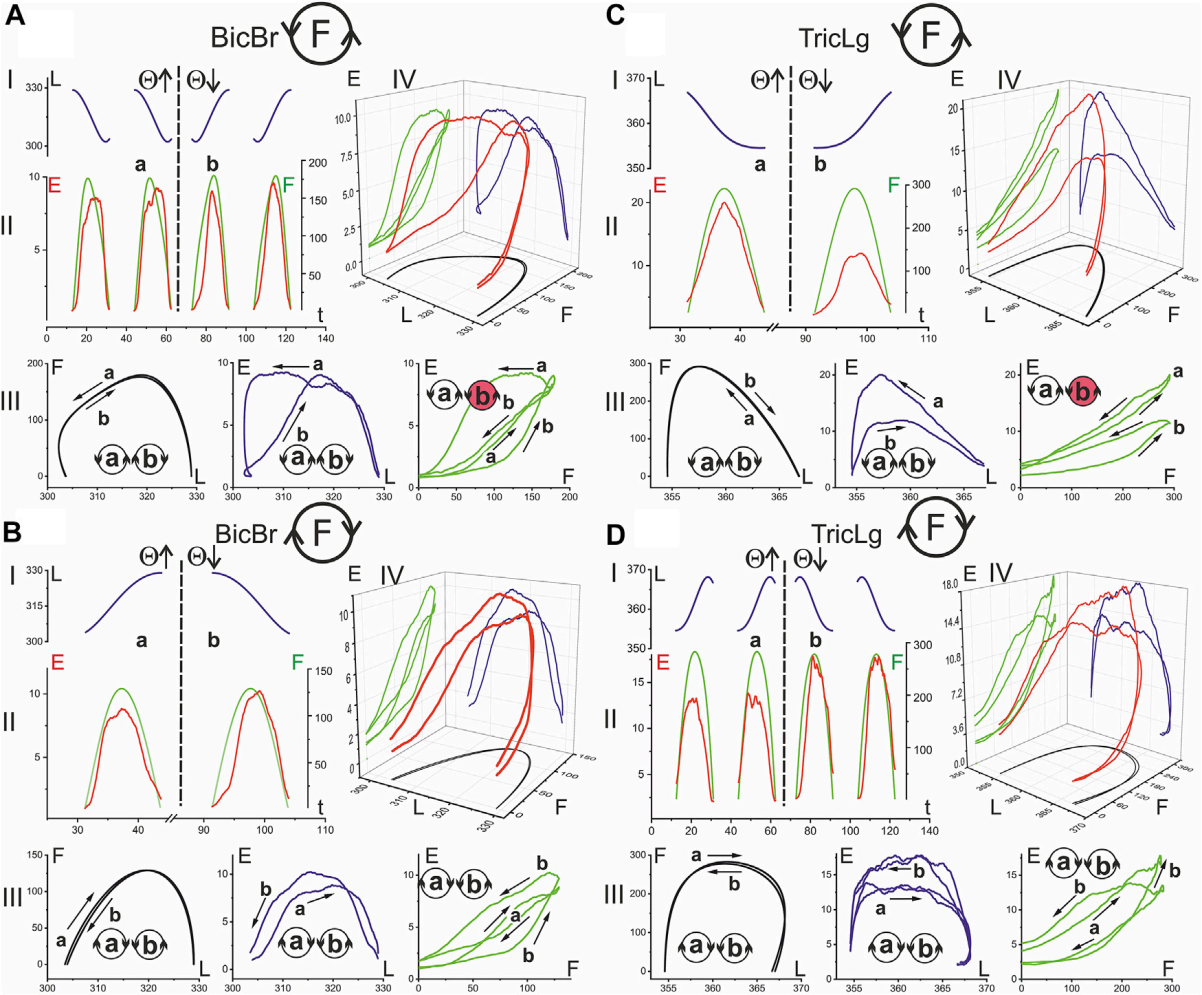
The hysteresis effects in the activity of the elbow muscles: relationship between EMG intensity (E), muscle length (L) and force (F). The data were obtained of subject YK with identified bone geometry, repetition of six identical tests for averaging procedure. The analysis is based on the segments of time records *L(t)* (blue)*, E(t)* (red)*,* and *F(t)* (green)*,* which are allocated strictly within the action zones of the forces generated by the muscle [Panels I, II in **(A–D)**]. After reordering the functional dependencies between variables, the 3D traces of the EMG intensity changes are presented in dependency on the respective mechanical parameters of the muscles *E(L, F)* (red color) [panel IV in **(A–D)**]. Functional dependencies *F (L), E (L),* and *E (F)* are defined as projections of three-dimensional traces on coordinate plates, and they are displayed in the respective colors: black, blue, and green [panels III in **(A–D)**]. Other designations: the circle with letter F inside designates the force direction in respective movement tests **(A–D)**; a and b are the phases of the test movement coinciding with rising (Θ↑) and falling (Θ↓) branches of the turning angle change; the circles with letters a and b inside designate the directions of the respective hysteresis loops (the directions mainly coincide for different pairs of variables with the exception of those marked in red). A continuation of the presentation of this experiment for the shoulder muscles is shown in Figure 6.

**FIGURE 6:**
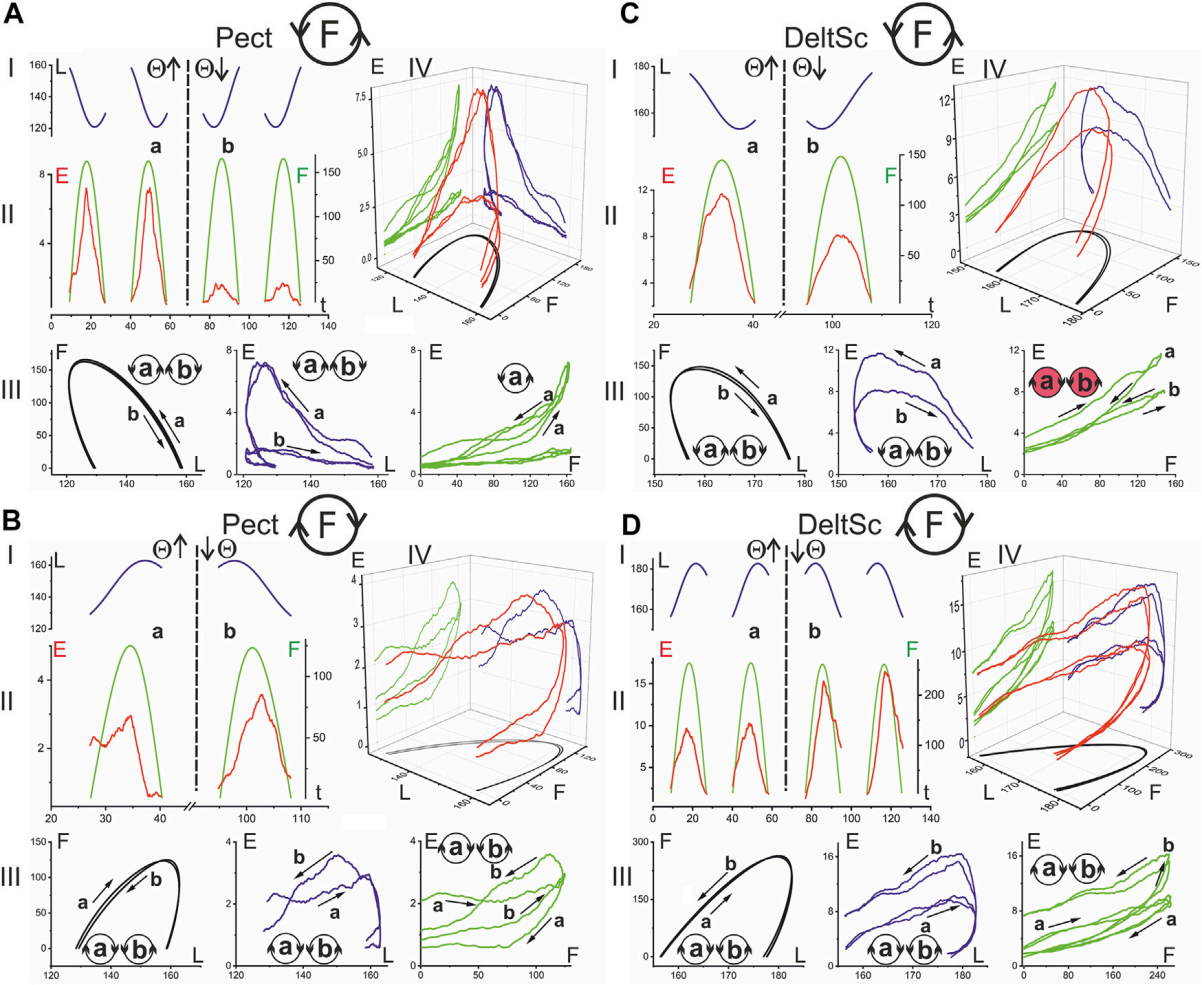
The hysteresis effects in the activity of the shoulder muscles. The data were obtained in the same experiment as in Figure 5; combining both drawings proved impractical due to their scope. The structure of the plots and all the designations (except for the names of muscles) are the same as in Figure 5.

The EMG responses of muscles involved in two-joint movements are primarily dependent on the forces generated by the muscles; at the same time, they are strictly modified by movement parameters such as muscle length and velocity. Therefore, from a formal point of view, EMG can be represented as a function of the following two variables: the force generated by the muscle and its length, that is, E(F, L). A common way of presenting such data would be three-dimensional plots that allow the relationships between variables to be observed. Figures 5, 6 show sets of temporary records of the three variables L, F, E (Panels I, II) and their three-dimensional reconstruction E(F, L) (IV), extended by the corresponding projections onto the following three coordinate plates: F(L); E(L); E (F) (III). One flexor and one extensor muscle were selected for each joint; identical representations were considered separately for F(ccw) and F(cw) (pairs of sets: A–D in Figures 5, 6).

In the present study, rather slow movements were used, and one cycle was completed in 30 s. This was necessary to obtain a reasonably good visual tracking quality for the force commands and a decrease in the weight of the dynamic components in the EMGs. The three-dimensional records E(F, L) in Figures 5, 6 compare EMG responses to identical force waves developing in the muscle shortening and lengthening phases. All muscles presented in these figures clearly show a higher location of EMG traces, where muscle shortening predominates. This corresponds to curves I and II during the creation of F(ccw) and F(cw) forces, respectively. Such differences in positioning the “shortening” and “lengthening” EMG waves are clearly observed on both the flexor and extensor muscles of both joints. During the application of F (ccw) forces, the EMG waves in *Pect* associated with muscle lengthening significantly outweigh the rather weak responses associated with shortening phases (Figure 6A); this difference remains visible in the case of F(cw) (Figure 6B) but to a lesser extent. The direction of hysteresis is usually considered one of its main characteristics; the directions of the F(L) and E(L) loops always coincide for the corresponding movement directions T (ccw) (Θ↑) or T (cw) (Θ↓) and change to the opposite direction with the change in force direction (Figures 6A,B). This may indicate a high degree of interdependence of the force and EMG intensity; however, sometimes this interdependence is likely to be violated, and the E(F) loops change their direction in comparison with the F(L) and E(L) loops (see the red colored circles in Figures 5A,C, 6C). Sometimes this can be explained by a slowdown in the EMG recording at the very beginning of muscle reactions in relation to force waves (see E(t) and the corresponding E(F) records in Figures 5A,C). At the same time, the possibility of a redistribution of activity between different agonists and their cocontraction with antagonists exists, which will affect the recorded EMG reactions, making them less stable and predictable in comparison with the mechanical parameters L and F.

### Integration of EMG Intensity in Relation to Ascending and Descending Phases of Successive Force Waves

**FIGURE 7:**
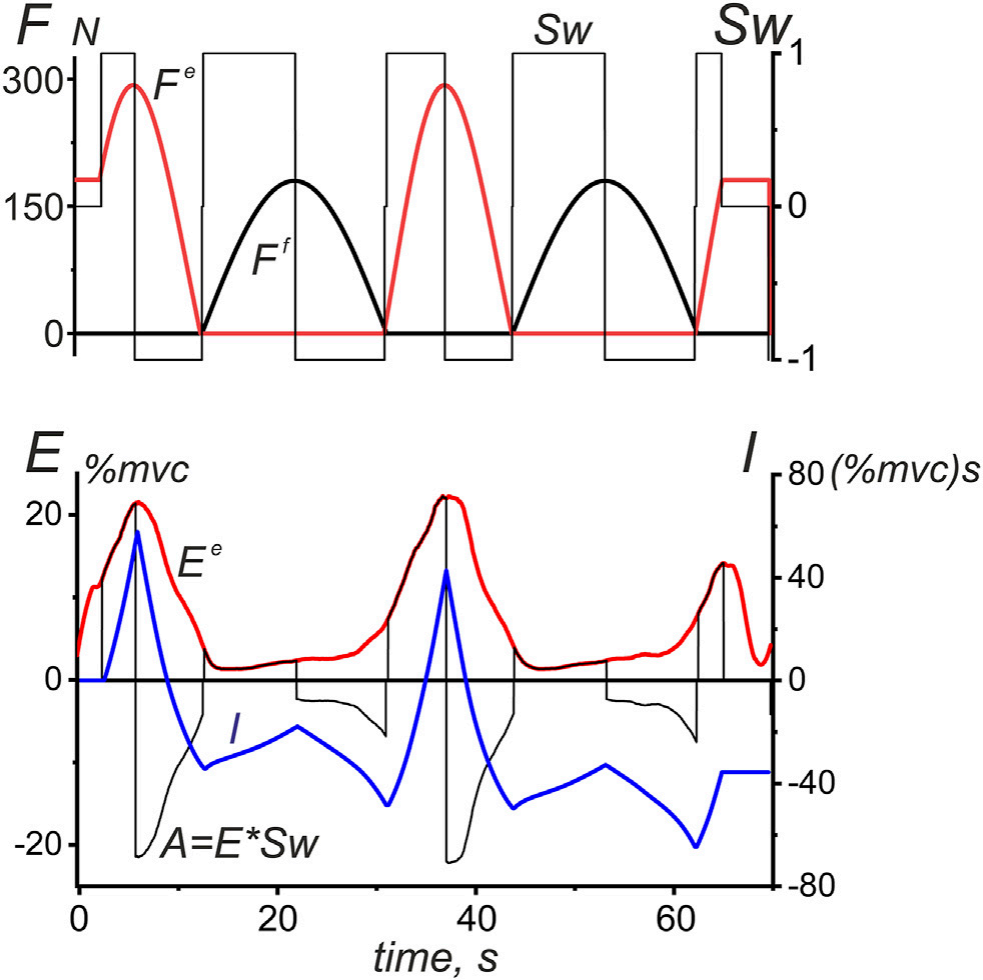
A procedure used to integrate the EMG intensity in the antagonist muscles of a given joint during the ascending and descending phases of successive force waves. An example is the EMG reaction of *TrLat* shown earlier in Figure 3. The switch function *Sw* at the upper plot is defined by Equation 3, and the integration procedure for the EMG intensity (*I*) is applied to the auxiliary function A = E*Sw; the integration result is an oscillatory process that correlates with the original EMG intensity curve, at the same time, stressing more distinctively differences between EMG areas at the ascending and descending phases of the force waves. The difference between the maximum and minimum points at each section of the integral curve (*I*), where this function changes monotonically, gives us a strict value of the EMG intensity area within the corresponding time intervals. The integral curve *I* makes it possible to more clearly represent the differences between the central commands acting on the studied muscles on the ascending and descending branches of force waves.

The use of the same IMP and ACP programs and the same positioning of the trajectory of movement relative to the axis of the shoulder joint provides a high degree of unification of data recorded from the same subject or even from different subjects with similar anthropometric parameters. This also applies to the calculated force waves in different agonist muscles, which for the most part can differ in amplitude, while having close values of the relative start and end times. Thus, the general analysis can be applied to EMGs recorded in different agonist muscles in different experiments. We propose the following approach for easy and simple averaging of EMG in all muscles of a given joint with the separation of their parts associated with successive ascending and descending phases of force waves in both agonist and antagonist muscles (Figure 7). The idea consists of a stepwise change of the sign of the EMG records at the descending phases of the force waves, which can be accomplished by multiplication of the EMG curve on the switch function (*Sw*) defined in the time or in the turning angle domains as follows:

$$Sw\left(t\right)=Sign\left(\frac{dF^{f}(t)}{dt}+\frac{dF^{e}(t)}{dt}\right),or\;Sw\left(\theta \right)=Sign\left(\frac{dF^{f}(\theta )}{d\theta }+\frac{dF^{e}(\theta )}{d\theta }\right),$$ (3)

where *F* ^*f*^ and *F* ^*e*^ are the forces in the flexors and extensors, respectively, and function Sign(x) is defined as follows:

$$Sign\left(x\right)=\{\begin{array}{c} 1,\;\;if\;x>0\;\\ 0,\;\;if\;x=0\\ -1,\;if\;x<0 \end{array}.$$ (4)

Multiplication of the corresponding EMG record ( E(τ) ) by the switch function ( Sw(τ) ) creates the sign-changing function Sw(τ)E(τ) that coincides with the original EMG in the intervals where the forces increase and change its sign to negative in the intervals where the forces decrease (Figure 7B) Integrating function Sw(τ)E(τ) over the time of movement, we obtain a variable function I(t) , in which the differences in the ordinates of neighboring switching points are equal to the EMG areas within the corresponding boundaries of the force change:

$$I\left(t\right)=\int^{t}Sw\left(\tau \right)E\left(\tau \right)d\tau .$$ (5)

**FIGURE 8:**
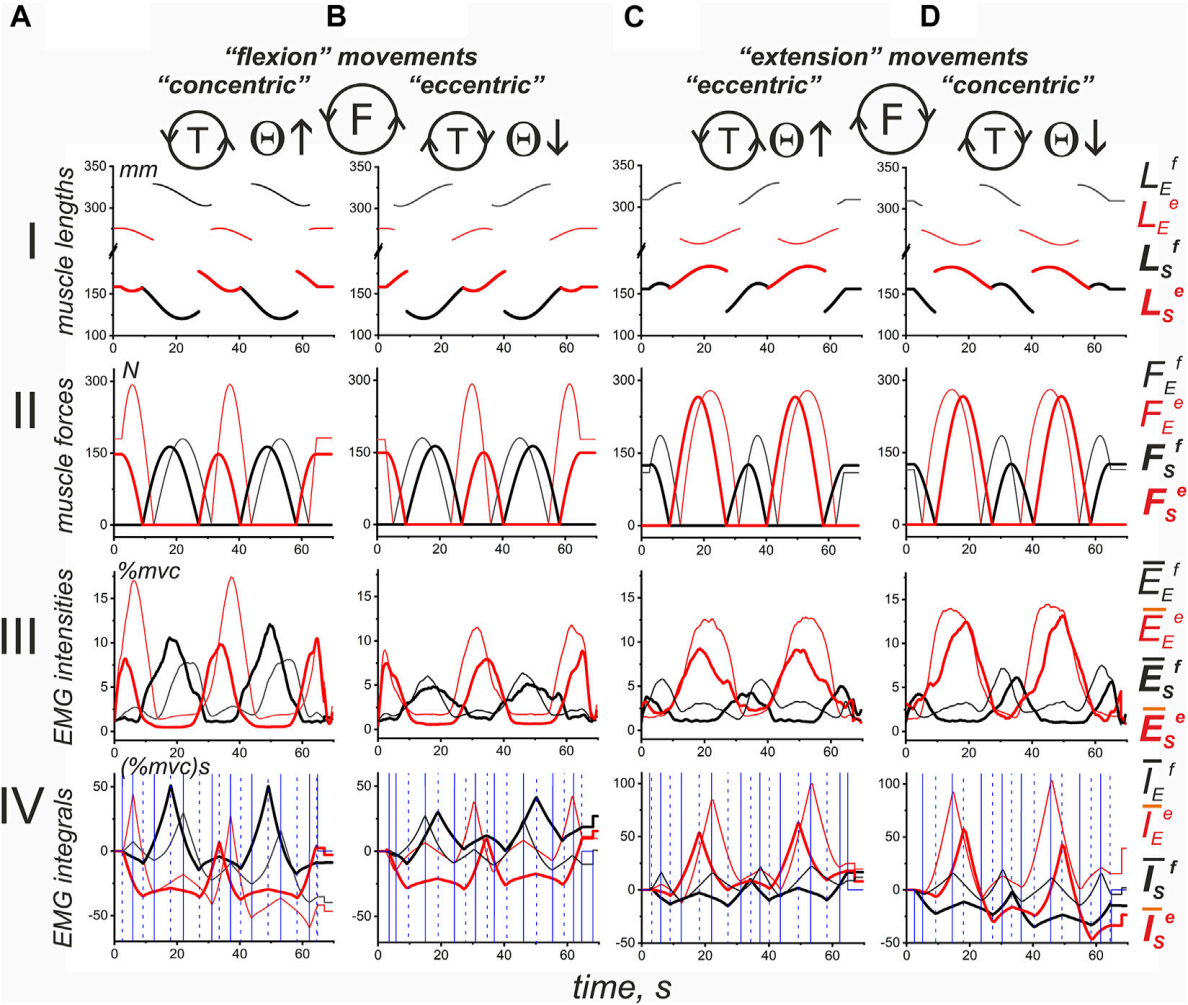
Average of the results obtained in identical experiments with four different subjects. The records of muscle length (I) and force (II) were taken from an experiment with subject YK, of which a complete anthropometric identification of his right arm was obtained (see Methods). First, for each subject, including YK, the average EMG recordings belonging to the following four muscle groups were obtained: **(A)** *elbow flexors* (an additional averaging procedure was applied to the records of *BrRad, BicBr, BicLg*); **(B)** *elbow extensors* (*TrBr, TrLat*); **(C)** *shoulder flexors* (*Pect, DeltCl*); and **(D)** *shoulder extensors* (*DeltSc*). Second, using the corresponding *Sw* functions reconstructed for the muscles of subject YK, the EMG integrals (as shown in Figure 7) were determined for each subject. Third, the corresponding EMG integrals, which were previously determined for different subjects, were averaged within each of the muscle groups, and the resulting records are presented in line IV. In addition, line III shows the results of the in-group averaging of the corresponding EMGs of the four subjects. The data related to flexor and extensor muscles are shown in black and red, where the thin and thick lines represent the muscles of the elbow and shoulder joints, respectively. The blue vertical lines in line IV correspond to the ascending and descending portions of the *Sw* functions defined for the muscles of the elbow (solid lines) and shoulder (dashed lines) joints.

**FIGURE 9:**
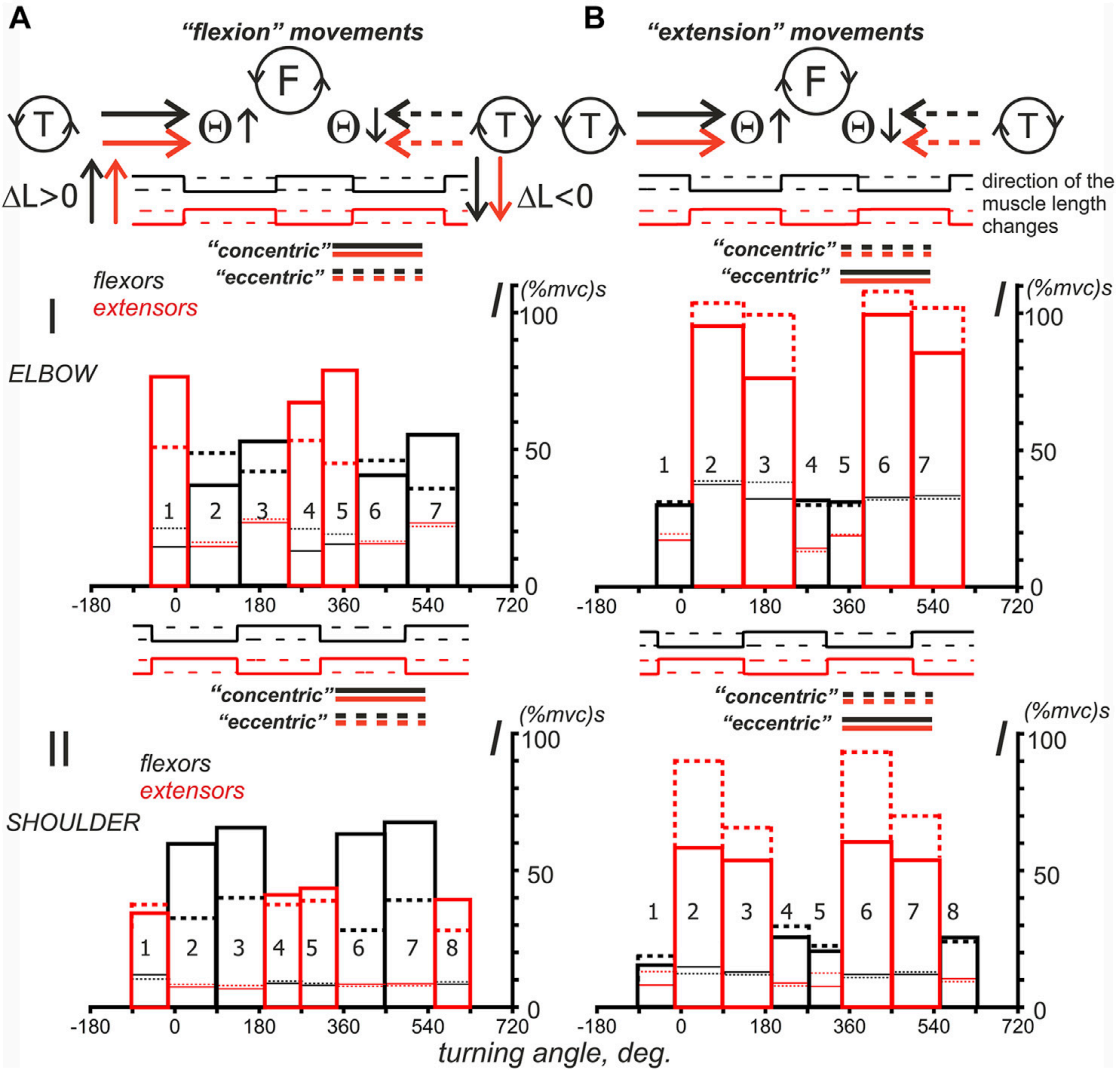
Turning angle-dependent distributions of the mean values of the average EMG integrals defined for four subjects in Figure 8. Two force direction **(A,B)** and two movement direction combinations are compared, as shown by the solid and dashed lines for the turning angle changes, i.e., T (ccw) and T (cw). The distributions of EMG integrals depending on the turning angle are presented for the sequences of intervals that correspond to the ascending and descending phases of the force waves, which are sequentially generated by the antagonistic muscles of the elbow and shoulder joints. Note the difference between the positions of the ascending and descending parts of the force waves for different force directions. Using the sequential numbering of the column bars in the Panel, the ascending parts correspond to the even numbers in F (ccw) and odd numbers in the F (cw) directions of force. The details of the data collection and calculation methods are presented in Figures 7, 8. The thick bars, which are marked by color (black—flexors, red—extensors), are related to the integral characteristics defined within the “own” force wave sectors, whereas the thin lines of respective color describe the effects of cocontraction within sectors with predominant activation of the antagonistic muscles; the solid and dashed lines refer to the turning angle change directions, as shown at the top of the figure.

Thus, we can obtain direct information about the EMG intensity of a given muscle, not only for those parts of the movement traces where it generates force but also for the areas of “responsibility” of the antagonist muscles. It seems to be important that the proposed approach makes it possible to apply the averaging procedure to the data obtained from different agonist muscles in different subjects (Figures 8, 9). However, such data are not suitable for exhaustive statistical analysis; we present them only to create a general impression about the EMG distributions for various movement patterns.

### Intragroup Averaging of the Results Obtained on Different Subjects

Figure 8 shows a possible application of the approach described above to jointly examine EMG components in different subjects. The mechatronic device and visual feedback allowed high-quality repetition of the experimental conditions for different subjects. The main problem of applying the method described above to different subjects is the presence of possible force wave shifts in accordance with the anthropometric parameters of the subject’s arm (SE and EH arm segments in Figure 1). We chose a group of four subjects, which also included subject YK with tomography and 3D printing of his bones, as well as the identification of the corresponding places of origin and insertion of his muscles. The subjects of the group had fairly close external anthropometric parameters, so the displacement of the force waves in the group did not exceed 3–6° in the cycle of movements.

Figure 8 shows the length and force records of the muscles of subject YK (Lines I, II), the average EMGs, defined for respective muscles within the group of subjects (III), and the corresponding EMG integrals (IV), defined as explained in Figure 7. The joint presentation of “in-group” EMG responses in four functional muscle groups can probably illustrate the common features of central commands accompanying the same movements performed by different people.

A shared consideration of the average EMG intensities and their phase-dependent integrals (lines III and IV in Figure 8) demonstrates a closeness in the positioning of reactions of muscles of the same functional groups belonging to different joints. Both the force waves and respective EMG components (Figures 8A–D, lines II, III) correlate with each other in both muscle groups of both joints. At the same time, one can observe a clear difference in the durations of the corresponding waves and their amplitudes associated with the force direction. For F(ccw), these waves are larger in flexors (Figures 8A,B), F(cw), and extensors (Figures 8C,D). Thus, when performing circular two-joint arm movements, the dominant types of movements can be distinguished, such as “flexion” and “extension,” in which the corresponding muscles of both joints are involved. In the flexor muscles, both the EMG intensity and their phase-dependent integrals are greater during the “flexion” movements; in the extensors, these parameters are higher during the “extensor” movements. On the other hand, “flexion” and “extension” movements are also clearly divided into “concentric” and “eccentric” subtypes, depending on the direction of the change in muscle length (see Figure 8, Line I). For flexor and extensor muscle groups, “concentric” movement subtypes correspond to the case when the directions of force and movement coincide, that is, F(ccw)-T(ccw) for flexors and F(cw)-T(cw) for extensors (Figures 8A,D). In contrast, opposite directions are associated with “eccentric” movements, as follows: F(ccw)-T(cw) (flexors) and F(cw)-T(ccw) (extensors) (Figures 8B,C). Consequently, the central commands to the muscles involved in cyclic arm movements are highly dependent on a combination of force and movement directions. Note that quotation marks were used around the terms: *flexion, extension, concentric,* and *eccentric*, as they refer to a full circular test movement or its part. At these time intervals, the introduced characteristics of movement prevail; however, there are also components of the opposite direction, but with a shorter duration.

The histograms in Figure 9, which represent the intra-group averaging of EMG integrals, are not statistical descriptions of the central commands that control similar movements in different subjects, but they can give us some preliminary information for further analysis. Despite the coincidence of the angular position of different phases of activity of the flexors in the “flexion” movements and extensors in the “extension” movements, the alternation of the ascending and descending phases of forces is opposite for them. For example, the areas of activity of the flexors of both joints in “flexion” movements, designated by numbers 2 and 6, correspond to the phases of increasing force (Figures 9AI,II), while the same numbers for the “extension” movements are associated with phases of decreasing force in the extensors (Figures 9BI,II). Such a difference reflects an opposite relationship between the “concentric” and “eccentric” directions in the “flexion” and “extension” movements.

It seems that the graphs presented in Figure 9 allow us to draw a conclusion about the possible differences that may exist between the activities of the muscles belonging to the elbow and shoulder joints. It partly concerns the EMG intensities in cocontraction phases with the muscles-antagonists (thin lines at the corresponding bars presented by thick lines). In the shoulder muscles, the cocontraction EMG intensities are minimal and lie in the range of 7.3–12.6 and 7.5–15.2 (%mvc) s for the F (ccw) and F (cw) force directions, respectively. In the elbow muscles, these components are much more intense, achieving values in the range of 12.4–24.3 and 13.4–39.1 (%mvc)s (Figures 9AI,II). Moreover, in the case of “extending” movements, the “coactivation” EMG intensities in the elbow flexors may approximately coincide with their basic levels (thick solid and dashed bars) or even exceed them (Figure 9BI **,** compare the thick and thin lines of black color in bars 1, 4, 5 and 2, 3, 6, 7).

**FIGURE 10:**
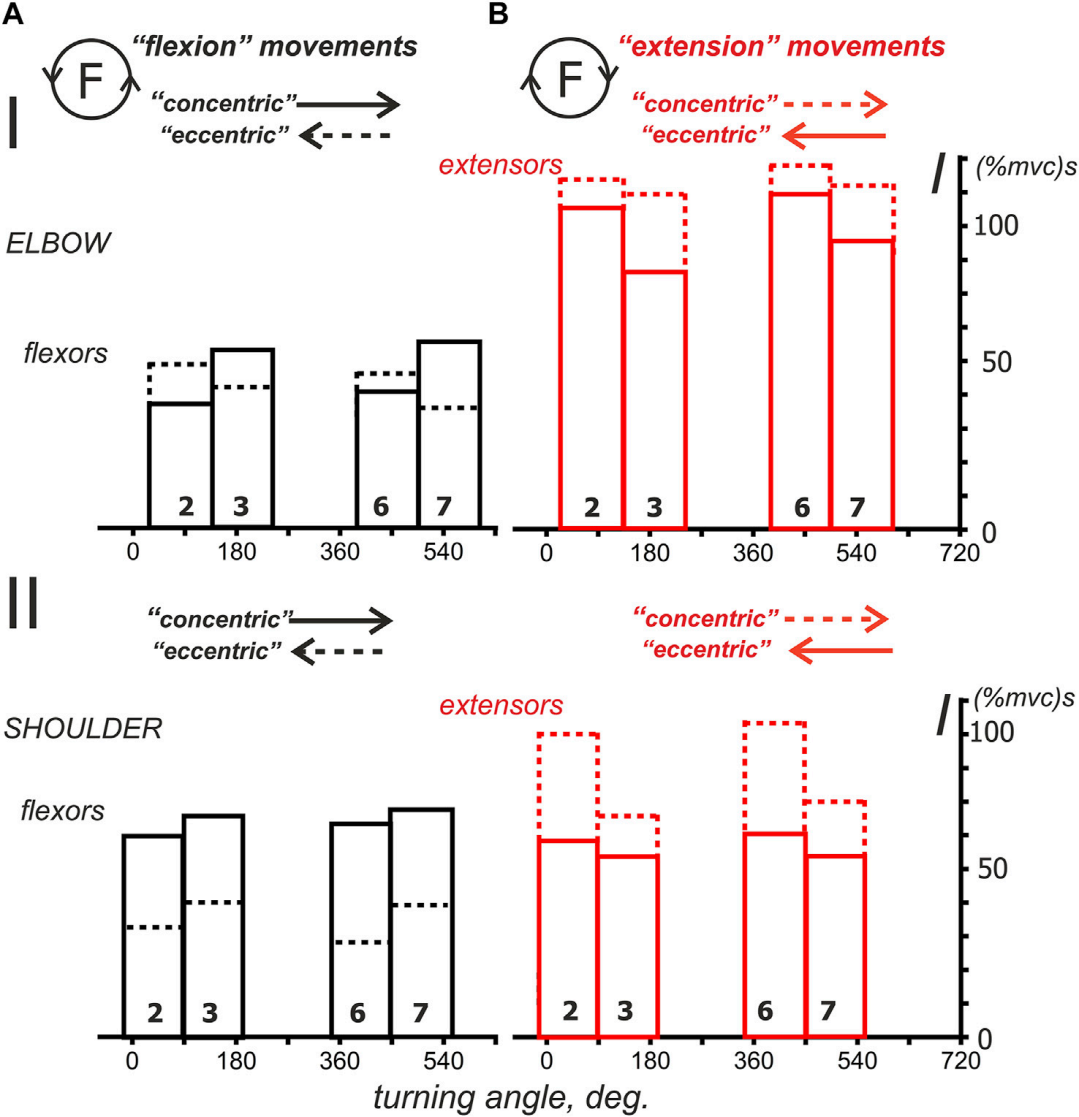
The average integral EMG characteristics considered for predominantly activated muscles during the “flexion” **(A)** and “extension” **(B)** movement paradigms. These reduced plots are extracted from the corresponding diagrams in Figure 9; the reactions of the antagonist muscles in these plots (extensors in A; flexors in B) are hidden for simplicity. Note the rotation of the “concentric” and “eccentric” directions for the flexor and extensor muscles.

To more clearly represent the general nature of probably associated with a stable increase in EMG responses of both flexors and extensors during the descending phases of force waves in the “concentric” subtypes of the “flexion” and “extension” movement patterns, for flexors, this coincides with the larger amplitudes of Columns 3 and 7 compared to Columns 2 and 6 (solid lines); for the extensors, the reverse order is clearly visible, i.e., Columns 2 and 6, which are represented by dashed lines, are higher than the similar Columns 3 and 7 (note the reverse order for the ascending and descending phases of force in this case). However, for the “eccentric” movement subtypes, such an order of the differences between “concentric” and “eccentric” subtypes of movement, we only have the corresponding columns in the graphs in Figure 10 left. As might be expected, “concentric” muscle contractions predominantly require more intense efferent influx than “eccentric” contractions, but this rule is not so obvious for the elbow flexors. We would like to note that in most cases, the extensor muscles are likely to be activated more intensely during the “extension” movements than the flexors during the “flexion” movements (compare Parts A and B in Figure 10); the muscles of the elbow joint, the difference can be up to two times greater. Another purely qualitative observation that follows from Figure 10 is only valid for the elbow flexors, when the dashed Columns 2 and 6 are higher than the similarly marked Columns 3 and 7 (Figures 10AI). In the elbow extensors, as well as in the flexors and extensors of the shoulder, the reverse order of the relationship between the components can be observed. For extensors, the last statement means decrements of the solid red Columns 3 and 7 with respect to similarly marked Columns 2 and 6.

### Statistical Analysis of the Average EMG Components in Subjects With Identified Arm Anthropometry

**FIGURE 11:**
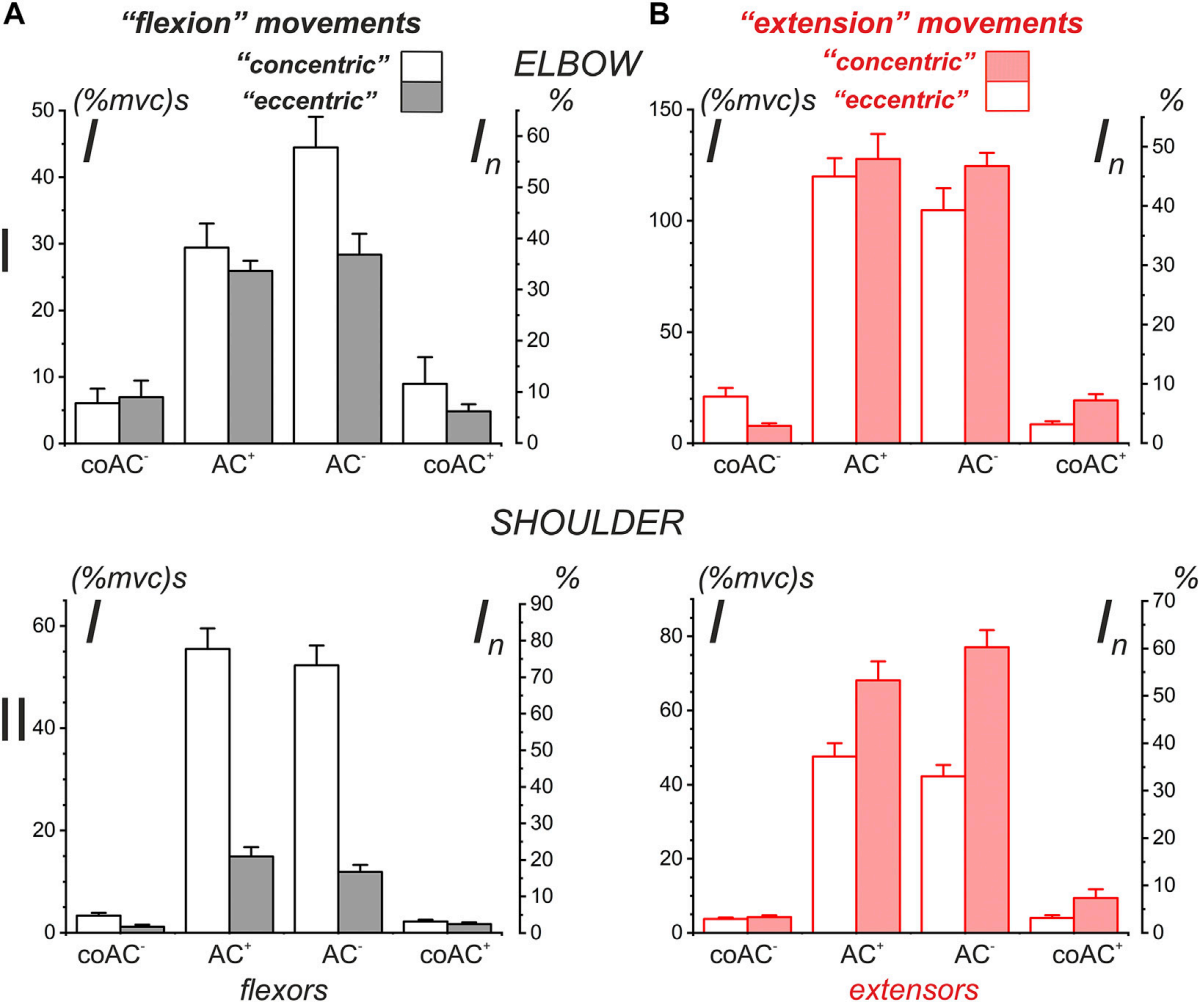
Statistical analysis of the average EMG components recorded in four different experiments on a subject with identified bone geometry (YK). This is a partial representation in graphical form of the quantitative data from Table 1. The additional scales *I*_*n*_ (%) on the right side of the plots represent the percentage normalization of the statistical parameters with respect to the sum of the means of all presented components in the plots. Note that the order of components in the plots along their *X*-axes coincides with the sequence of their appearance in the corresponding test movements.

To test the relationship between the various EMG components in the muscles of both joints in “flexion” and “extension” movement patterns, we applied statistical analysis to data from four identical experiments performed on the same subject YK with arm anthropometry (Table 1; Figure 11). In general, this analysis is consistent with the above qualitative study of test movements in different people (Figures 9, 10). We were unable to confirm our preliminary conclusions about the existence of a clear difference between the central commands in the “concentric” and “eccentric” modes of movement. Such a difference can be visually observed for all muscle groups, considering only the amplitudes (mean values) of the corresponding columns in Figure 11. However, the statistical significance of these differences was confirmed only for both types of shoulder muscles and partly for the elbow flexors (highlighted cells in Table 1).

**TABLE 1:** Statistical analysis of the average EMG components recorded in four different experiments on a subject YK with identified bone geometry.

Joint	Muscles	Movement direction	AC	AC^+^	AC^−^	t-test: AC^+^-AC^−^	coAC^+^	coAC^-^	t-test: coAC^+^-coAC^−^
**Elbow**	**Flexors**	CCW	73.9 ± 8.1	29.4 ± 3.6	44.5 ± 4.5	**t** = **2.578**	8.9 ± 4.0	6.1 ± 2.2	t = 0.638
									DF = 16
			*n* = 18	*n* = 18	*n* = 18	**DF** = **34**	*n* = 9	*n* = 9	*p* = 0.535
						***p*** = **0.014**			
		CW	54.3 ± 4.4	25.9 ± 1.5	28.3 ± 3.1	t = 0.687	4.8 ± 1.0	6.9 ± 2.5	t = 0.771
									DF = 16
			*n* = 18	*n* = 18	*n* = 18	DF = 34	*n* = 9	*n* = 9	*p* = 0.458
						*p* = 0.496			
		t-test: CCW-CW	**t** = **2.127**	t = 0.883	**t** = **2.90**		t = 0.992	t = 0.271	
								DF = 16	
				**DF** = **34**	DF = 34		**DF** = **34**			DF = 16
				***p*** = **0.041**	*p* = 0.382		***p*** = **0.006**	*p* = 0.347		*p* = 0.790
	**Extensors**	CCW	225.4 ± 17.7	128.2 ± 11.3	105.1 ± 9.9	t = 1.180	8.5 ± 1.3	21.1 ± 3.8	**t** = **3.082**
						DF = 30			**DF** = **14**
			*n* = 16	*n* = 16	*n* = 16	*p* = 0.246	*n* = 8	*n* = 8	***p*** = **0.014**
		CW	253.2 ± 16.9	128.2 ± 11.3	125.1 ± 5.9	t = 0.247	19.3 ± 2.8	7.7 ± 1.2	**t** = **3.755**
									**DF** = **14**
			*n* = 16	*n* = 16	*n* = 16	DF = 30	*n* = 8	*n* = 8	***p*** = **0.002**
						*p* = 0.806			
		t-test: CCW-CW	t = 1.132	t = 0.560	t = 1.728		**t** = **3.445**	**t** = **3.309**	
				DF = 30	DF = 30		DF = 30	**DF** = **14**		**DF** = **14**
				*p* = 0.266	*p* = 0.579		*p* = 0.094	***p*** = **0.004**		***p*** = **0.010**
	**Shoulder**	**Flexors**	CCW	108.9 ± 7.7	56.1 ± 4.1	52.8 ± 3.9	t = 0.575	2.2 ± 0.3	3.3 ± 0.6	t = 1.745
DF = 10									
*n* = 12				*n* = 12	*n* = 12	DF = 22	*n* = 6	*n* = 6	*p* = 0.121
						*p* = 0.570			
CW			27.1 ± 3.3	15.1 ± 1.8	12.0 ± 1.4	t = 1.300	1.6 ± 0.3	1.1 ± 0.4	t = 1.047
			*n* = 12	*n* = 12	*n* = 12	DF = 22	*n* = 6	*n* = 6	DF = 10
						*p* = 0.206			*p* = 0.320
t-test: CCW-CW			**t** = **9.701**	**t** = **9.160**	**t** = **9.747**		t = 1.227	**t** = **3.184**	
				**DF** = **22**	**DF** = **22**		**DF** = **22**	DF = 10		**DF** = **10**
				***p*** = **0.000**	***p*** = **0.000**		***p*** = **0.000**	*p* = 0.248		***p*** = **0.010**
		**Extensors**	CCW	90.8 ± 6.5	48.1 ± 3.7	42.7 ± 3.1	t = 1.121	4.0 ± 0.8	3.8 ± 0.3	t = 0.258
							DF = 22			DF = 10
				*n* = 1	*n* = 1	*n* = 1	0.274	*n* = 6	*n* = 6	*p* = 0.802
	CW		146.6 ± 8.8	68.8 ± 5.2	77.8 ± 4.7	t = 1.287	9.4 ± 2.3	4.2 ± 0.5	t = 2.105
									DF = 10
			*n* = 12	*n* = 12	*n* = 12	DF = 22	*n* = 6	*n* = 6	*p* = 0.062
						*p* = 0.211			
	t-test: CCW-CW		**t** = **5.095**	**t** = **3.252**	**t** = **6.271**		t = 2.139	t = 0.763	
			**DF** = **22**	**DF** = **22**	**DF** = **22**		DF = 10	DF = 10	
			***p*** = **0.000**	***p*** = **0.003**	***p*** = **0.000**		*p* = 0.058	*p* = 0.465	

A two-sample *t* test was used to determine the statistical significance of the difference between the respective parameters, and the highlighted cells indicate a statistically significant difference at the *p* < 0.05 level. The analysis concerns the “flexion” and “extension” movement patterns described in Figure 11. Four components of EMG activity in the flexor and extensor muscle groups are considered. The active components AC^+^ and AC^−^ represent the EMG activity, which coincides in time with the phases of the active muscle contraction during generation of the respective force waves, while the coAC^+^ and coAC^−^ components reflect their activity that opposes the active contractions of the antagonist muscles. The superscripts indicate correspondence of the components to the ascending (+) and descending (-) phases of the force waves. Additionally, we also considered the distribution of the sum of AC^+^ and AC^−^ components (AC). The components are presented in (*mvc%*) *s*; they are determined by multiplying the mean intensities of the EMG sections recorded in the corresponding time intervals by the duration of the intervals. The mean EMG intensity values (m *±* mse) were defined for the muscles of a given group (the grouping is described in Figure 8) in different experiments.

Statistical analysis made it possible to confirm some interesting features of EMG coactivation components in the elbow extensors during “extensor” movements. If we return to Figure 3 and examine the superimposed EMG records from both heads of triceps, presented as the dependences on the turning angle (red traces on the right side of Figures 3D,E), then a striking coincidence regarding the EMGs for opposite directions of movement could be noticed, in which the ascending and descending phases of force change their order. This overlap seems to be associated with the respective asymmetry of the coactivation areas of the EMGs. The evident asymmetry of the coactivation components of EMGs in the elbow extensors is supported statistically (*t*-test: coAC^+^- coAC^−^ in Table 1). Such positional modification of the central commands to the elbow extensors is visible not only during periods of joint activation with the flexors but also during active force waves in these muscles. The EMG waveforms in these cases are clearly asymmetric, and the asymmetry remains much the same for the opposite direction of movement (Figures 3D,E). At the same time, the EMG waveforms seem to be rather complex, so the asymmetry between their branches, corresponding to the ascending and descending components of the force waves, has not been statistically confirmed (*t*-test: AC^+^ - AC^−^ in Table 1).

By comparing the muscles of the two joints, a clearer and more predictable difference can be observed between the central commands of the shoulder muscles in the “concentric” and “eccentric” modes of movement (see Lines *t*-test: CCW-CW in Table 1). The *Pect* and *DeltSc* muscles belonging to the shoulder joint usually demonstrate highly symmetric EMG reactions (Figures 4A,B), and statistical analysis confirmed the absence of asymmetry for all EMG components (*t*-test columns in Table 1). In addition, we would like to stress that the *DeltCl* reactions were usually more unpredictable than those of *Pect* and *DeltS*, which may be related to its mixed nature with the presence of both flexor and extensor components (Figure 4C).

## Discussion

The present study demonstrates progress in most of the methodological aspects, allowing one to abandon the use of more traditional mechanical installations when organizing test movements and external loads. We have developed a computerized mechatronic system for studying forced planar movements of the forearm in direct connection with the creation of a target force by the subject’s hand in the visual feedback mode (Zasada et al., 2020). In addition, we applied three-dimensional identification of the bones of the subject’s arm with the sites of origin and insertion of the muscles under study, which increased the accuracy when determining the temporal changes in their forces and lengths (Gorkovenko et al., 2020). This made it possible to obtain a more detailed description of the average EMGs recorded from the elbow and shoulder muscles. Together with the traces E(L, F), recorded in the form of three-dimensional curves (Figures 5, 6), we also obtained the opportunity to fix their projections onto the coordinate planes. It is known that the main features of nonlinear effects in active muscles are closely related to the direction of the hysteresis loops recorded in the main modes of contraction (for review see: Kostyukov, 2007). For all combinations of force and length direction changes, our study showed the coincidence of the directions of loops F(L), E(L), and E(F) in three quarters of all cases, including complete coincidence for the first two types of dependencies (Figures 5, 6). It seems likely that despite the well-known type of nonlinear relationships between efferent signal, force, and length, which initially exist in isolated muscles themselves (Kostyukov 1987, 1998), their mutual forced interaction within a system of many muscles can sometimes distort this relationship.

Experimental studies of the central processes associated with the interaction of torques acting around various joints during multijoint movements have been limited mainly to self-initiated reaching movements (Almeida et al., 1995; Gottlieb 1998; Gribble and Ostry 1998; Galloway and Koshland, 2002; Maeda et al., 2017). In these studies, attention is focused on single-joint movements, provided that the other involved joints can move freely. It has been established that muscles spanning joints that are adjacent to the moving joint contract before movement onset, therefore compensating for the interaction torques. Such compensation has been demonstrated for shoulder muscles during pure elbow movements (Almeida et al., 1995; Gribble and Ostry 1998; Galloway and Koshland 2002) and for elbow muscles during pure shoulder movements (Almeida et al., 1995; Galloway and Koshland 2002). Obviously, the analyses in these studies are focused on fast dynamic processes rather than the slow, quasi-static effects of muscle interactions that are the subject of this study.

The division of circular movements into “*flexion*” and “*extension*” types according to the predominant type of activity along the complete cycle of movement was introduced to simplify the EMG analysis in relation to the ascending and descending phases of force waves. A more precise determination of muscle length changes by anthropometric identification (Gorkovenko et al., 2020) showed that the extreme points of length changes lie close to the boundaries of the corresponding force waves (Figures 2–4); therefore, for simplicity, the force waves might be considered only in association with the prevailing direction of movement. This allows us to consider the “*flexion*” and “*extension*” movement types in a simplified form by dividing them into “*concentric*” and “*eccentric*” (Figures 8–11). Neuromuscular studies have shown that muscles have strong contractile property asymmetry, which depends on the length of the muscle and the level of efferent activity, as well as on the direction of their change (for a review, see: Kostyukov, 2007). An individual muscle needs a higher intensity of efferent activation during concentric contractions compared to eccentric contractions; therefore, in the first case, a higher level of EMG intensity can be expected. Likewise, the differences between the “*concentric*” and “*eccentric*” subtypes should be manifested in both the “*flexion*” and “*extension*” types of two-joint movements.

The division of circular movements into “*flexion*” and “*extension*” types follows from the separation of the synergistic effects on the *coinciding* and *opposing* types (Kostyukov 2016; Kostyukov and Tomiak, 2018). The *coinciding* synergy corresponds to simultaneous loading of muscles belonging to different joints and having the same modality (flexors-flexors; extensors-extensors), while the o*pposing* synergy describes combinations of muscles with different modalities (flexors-extensors; extensors-flexors). Based on a purely geometrical consideration, it was shown that for both circular and linear movement types, *coinciding* synergy prevails; in the case of circular movements, there are two segments of *coinciding* synergy with different sizes with respect to the entire trace length. Longer segments of the *coinciding* synergy are more distally located, and their functionality, which determines the choice of preferential activation between flexors and extensors, depends only on the direction of the generated force. It can be noted that the proposed division of movements into such types refers to their more distal parts with a convex shape, while the narrower segments of the *coinciding* synergy with the simultaneous activation of the corresponding antagonist muscles are concave. The distance and curvature factors of movement tracks may have been important for the formation of central commands coming to muscles, but at present, such issues have not been considered.

Differences between “*concentric*” and “*eccentric*” activation patterns were clearly visible in the shoulder muscles, where they were not only recorded for full EMG waves but also persisted for separate components associated with different phases of the force change. This was qualitatively demonstrated by intragroup averaging in a group of four different subjects, and these results were confirmed by statistical processing of four experiments with the subject with anthropometric identification of his arm parameters. Similar differences between “*concentric*” and “*eccentric*” movements are also observed for the muscles of the elbow joint when considering only the corresponding mean parameters of different EMG components (Figures 10, 11); however, the statistical significance of these differences was only partially confirmed for the elbow flexors (highlighted cells in Table 1). We would also like to emphasize that the elbow muscles, both flexors and extensors, show a high degree of variability of the incoming central commands. It can be assumed that one of the mechanisms of the insignificant difference between “*concentric*” and “*eccentric*” reactions of the elbow muscles may be associated with a redistribution of activity between agonists; this may also be due to the rather large weight of the cocontraction EMG components in these muscles (Figures 9, 11). Sometimes, in single experiments, we observed the appearance of significant cocontraction EMG components that are compatible in amplitude with the main activation components (e.g., *BicLg* reactions in Figure 3C). Moreover, in the case of the elbow joint, a situation can become more complicated because some of its muscles are biarticular (for example, *BicBr* and *TrLg*) (Van Bolhuis et al., 1998). On the other hand, information on EMG is inevitably rather limited due to the inaccessibility of deep muscle tissue in the experiment and incomplete electrode coverage.

The observed differences between the patterns regarding the central commands to the muscles belonging the proximal (shoulder) and distal (elbow) joints might be at least partially consistent with the “leading joint” hypothesis proposed by Dounskaia for the analysis of multijoint movements (Dounskaia, 2005; Dounskaia and Wang, 2014). This hypothesis suggests that complex multijoint movement can be simplified for analysis by choosing the proximal, so-called “lead joint” for initial consideration. When comparing the EMG patterns in the shoulder and elbow muscles in our experiments, it follows that the shoulder muscles, as they belong to the “leading joint”, exhibit more “predictable” behavior. First, these responses may be more clearly distinguished as “*concentric*” and “*eccentric*” with respect to the direction of movement. Second, the shoulder muscles have a pronounced symmetry regarding the EMG responses with respect to the ascending and descending phases of the corresponding force waves, which noticeably distinguishes them from the elbow muscles. Third, the muscles of the shoulder demonstrate a more “economical” nature of central activation in comparison with the elbow muscles due to the lesser degree of cocontraction activity. Most often, the coactivation of antagonist muscles is considered one of the mechanisms that can increase joint stiffness, which may be important for multijoint movements (Kearney and Hunter, 1990; Hill et al., 2008; Hirai et al., 2015). On the other hand, the simultaneous contraction of antagonists can also have a profound stabilizing effect on the motor control system, reducing the so-called uncertainty effects associated with muscle hysteresis (Kostyukov, 1998, 2007; Gorkovenko et al., 2012).

In various human movements, three partially independent types of muscle synergy are usually considered. *Kinematic synergy* is represented by covariances between simultaneous changes in joint angles (Santello and Soechting, 2000); it has been used to describe distal movements such as different types of manual exploration (Thakur et al., 2008) and typing (Soechting and Flanders, 1997). *Force (kinetic) synergy* is described in the forced contractions of distal hand muscles during grip and voluntary interaction of the fingers (Santello and Soechting, 2000; Grinyagin et al., 2005). *Activation (muscle) synergy* is explored, for example, during static positioning of the hand (Weiss and Flanders, 2004; Castellini and van der Smagt, 2013) or during creation of active forces by the fingers of the hand (Valero-Cuevas, 2000; Latash et al., 2007). The *activation synergies* are also studied in real multijoint motor programs, such as locomotion (Ivanenko et al., 2004; Wang et al., 2013). Due to the great number of muscles involved in these motor acts, the synergetic effects are studied using multichannel EMG records and special correlation computing procedures, such as primary component analysis (Ivanenko et al., 2004, 2005). In recent years, these approaches have also been applied to the analysis of targeted movements of the forelimbs in humans (Steele et al., 2015; Gorkovenko et al., 2019, 2020; Turpin et al., 2020). In the above investigations and in the present study, slow arm movements that do not involve distal hand muscles are considered. Therefore, it seems sufficient in this case to consider only the *force* and *activation synergies*, ignoring the effects associated with *kinematic synergy*. The average EMG records, which are used to assess the *activation synergy*, show a strong dependence on the force changes; therefore, in this combination, the *force synergy* can be considered the primary, initial element, and the *activation synergy* is secondary. On the other hand, kinematic data, represented by muscle length trajectories, have strong modulating effects on *activation synergy*, dividing it into “*concentric*” and “*eccentric*” subtypes.

The forces generated by the muscles during two-joint movements are determined by the torques acting around the respective joints. During the entire period of cyclical movement, each of these torques consists of two waves of different signs and durations; one wave, which is positive, is generated by the flexors, and the other, which is negative, is generated by the extensors. The moments of switching between periods of antagonist muscle activity or the corresponding points on the trajectories of movement are primarily determined by the corresponding margins of the force waves; changing the direction of the end-point force changes the order of activation of the antagonist muscles. The positions of the switching points in the working area can be determined using a geometric modeling method that is suitable for both circular and linear movement trajectories (Kostyukov, 2016; Kostyukov and Tomiak, 2018). The positioning of these points depends on the lengths of the arm segments, which show a definite scatter for various subjects.

## Conclusion

The positional changes in the averaged EMGs of the elbow and shoulder muscles were compared for all combinations of direction of movement and generated force in two-joint circular arm movements. The averaged EMG traces in muscles of both joints show their close correspondence to the related force traces, however, the coactivation patterns of activity in agonists and antagonists were also often encountered. The EMG waves related to the respective force waves were strongly dependent on the predominant direction of the muscle length changes within the correspondent force wave locations: the EMG intensities were higher for the shortening muscle movements (*concentric* contractions) and lower during muscle lengthening (*eccentric* contractions). For the movements under study, it seems sufficient to consider only the *force* and *activation synergies*, ignoring possible effects associated with *kinematic synergy*. The average EMG records, which are used to assess the *activation synergy*, show a strong dependence on the force changes; therefore, in the pair, the *force synergy* can be considered the primary, initial element, while the *activation synergy* is secondary. On the other hand, kinematic data, represented by muscle length trajectories, have strong modulating effects on *activation synergy*, dividing it into “*concentric*” and “*eccentric*” subtypes.

## Data Availability

The raw data supporting the conclusion of this article will be made available by the authors, without undue reservation.

## Abbreviations

ACP, arbitrary contraction program; BicLg, m. biceps brachii caput longum; BicBr, m. biceps brachii caput breve; BrRad, m. brachioradialis; CNS, central nervous system; DOF, degree of freedom; EMG, electromyogram; F(ccw), force in counterclockwise direction; F(cw), force in clockwise direction; IMP, imposed movement program; Pect, m. pectoralis pars clavicularis; DeltSc, m. deltoideus pars scapularis; DeltCl, m. deltoideus pars clavicularis; TrLat, m. triceps brachii caput laterale; TrLg, m. and triceps brachii caput longum; MVC, maximal voluntary contraction; MMS, mechatronic mechanostimulator; T(ccw), turning in counterclockwise direction; T(cw), turning in clockwise direction.
